# Pharmacological Treatment of Fibromyalgia Syndrome: A Practice-Based Review

**DOI:** 10.1007/s11916-024-01277-9

**Published:** 2024-07-23

**Authors:** Valeria Giorgi, Piercarlo Sarzi-Puttini, Greta Pellegrino, Silvia Sirotti, Fabiola Atzeni, Alessandra Alciati, Riccardo Torta, Giustino Varrassi, Diego Fornasari, Stefano Coaccioli, Sara Francesca Bongiovanni

**Affiliations:** 1 Unità di Ricerca Clinica, Gruppo Ospedaliero Moncucco, Via Soldino, 5, 6900 Lugano, CH Switzerland; 2 Rheumatology Unit, IRCCS Ospedale Galeazzi Sant’Ambrogio, Milan, Italy; 3 Department of Biomedical and Clinical Sciences, University of Milan, Milan, Italy; 4 Rheumatology Unit, Department of Experimental and Internal Medicine, University of Messina, Messina, Italy; 5 Department of Clinical Neurosciences, Villa S. Benedetto Menni, 22032, Albese con Cassano, Como, Italy; 6 Humanitas Clinical and Research Center, Rozzano, 20089 Milan, Italy; 7 Clinical Psychology, Department of Neuroscience, University of Turin, Turin, Italy; 8 Paolo Procacci Foundation, Rome, Italy; 9 Department of Medical Biotechnology and Translational Medicine, Università degli Studi di Milano, Milan, Italy; 10 President, European League Against Pain, Zurich, Switzerland

**Keywords:** Fibromyalgia syndrome, Pharmacological treatment, Antidepressants, Anticonvulsants, Personalized therapy

## Abstract

**Purpose of Review:**

Fibromyalgia Syndrome (FMS) is a complex chronic pain condition characterized by widespread musculoskeletal pain and numerous other debilitating symptoms. The purpose of this review is to provide a comprehensive overview, based on everyday clinical practice, of the drugs presently employed in the treatment of FMS.

**Recent Findings:**

The treatment of FMS is based on a multimodal approach, with pharmacologic treatment being an essential pillar. The drugs used include tricyclic antidepressants, serotonin and noradrenaline reuptake inhibitors, other antidepressants, anticonvulsants, myorelaxants, and analgesics. The effectiveness of these medications varies, and the choice of drug often depends on the specific symptoms presented by the patient. Many drugs tend to either address only some domains of the complex FMS symptomatology or have a limited effect on pain.

**Summary:**

Each treatment option comes with potential side effects and risks that necessitate careful consideration. It may be beneficial to divide patients into clinical subpopulations, such as FMS with comorbid depression, for more effective treatment. Despite the complexities and challenges, the pharmacological treatment remains a crucial part for the management of FMS. This review aims to guide clinicians in prescribing pharmacological treatment to individuals with FMS.

## Background

Fibromyalgia syndrome (FMS) is a complex chronic pain condition. It's characterized by widespread musculoskeletal pain, profound fatigue, sleep disturbances, and numerous other symptoms affecting various organs and systems. These include dysautonomias, regional pain syndromes, and mood disturbances [1]. The latest diagnostic criteria have placed increased emphasis on non-musculoskeletal symptoms [2, 3], which are almost always present among patients, varying in number and severity [4]. 

The treatment of FMS is based on a multimodal approach to better address the array of symptoms experienced by patients. Pharmacologic treatment forms a crucial part of this approach. While it's often insufficient alone to control the complex symptoms of this syndrome, it can provide satisfactory results for some patients [4]. Three drugs, pregabalin, duloxetine, and milnacipran, have been FDA-approved for FMS treatment. Currently, no drugs are EMA-approved for this condition, making any drug use for FMS off-label. This review offers an overview of the drugs used in FMS treatment. While many of these drugs primarily serve as analgesics, it's crucial for clinicians to always bear in mind the nociplastic origin of FMS pain when prescribing pharmacological treatment to individuals with fibromyalgia [5].

## Pharmacological Treatment: General Aspects

FMS is a complex condition influenced by both peripheral and central factors [6]. Peripheral factors include an inflammatory environment and possibly autoantibodies against satellite glial cells [7]. Central factors involve altered neurotransmitter levels and brain activity in pain processing regions, highlighting central sensitivity's importance [8–11]. Effective FMS drugs mainly target the central nervous system (CNS), like antidepressants and anticonvulsants (Table 1). However, these treatments often fall short. Analgesics and non-steroidal anti-inflammatory drugs (NSAIDs) have proven ineffective, tricyclic antidepressants have limited effects, while duloxetine, milnacipran, and pregabalin have shown promise [12]. Data have not changed much since then, although there are some novelties.

**Table 1:** Medications used for different symptoms in FM

Drug	Pain	Sleep	Fatigue	Rigidity	Mood
TCAs	+	+	+	±	-
SSRIs	±	±	±	±	+
SNRIs	+	-	+	±	+
MAO-I	±	±	±	±	±
NSAIDs	-	-	-	-	-
Anticonvulsants	+	+	+	+	-
Sedatives/Hypnotics	-	+	-	+	-
Opioids	+	+	-	-	-
Myorelaxants	+	-	±	+	±

*SSRIs* Selective Serotonin Reuptake Inhibitors, *SNRIs* Serotonin and Norepinephrine Reuptake Inhibitors, *MAO-Is* Monoamine Oxidase Inhibitors, *NSAIDs* Non-Steroidal Anti-Inflammatory Drugs, *TCAs* Tricyclic Antidepressants

Prescribing medication for FMS is complex, and should follow the principle of "start low and go slow." Patients should discern between disease symptoms and medication side effects. Using multiple medications can address FMS's complex nature, and using symptom assessment scales like the Fibromyalgia Assessment Status (FAS) can monitor symptom intensity [13]. Management typically requires a multimodal approach, extending beyond medication use alone.

The following sections analyze various medications studied and commonly used in clinical practice.

## Antidepressants

The effectiveness of antidepressant drugs in treating chronic pain syndromes is now well-established. The type of chronic pain is important in this setting, since antidepressants are especially recommended for chronic primary pain syndromes [14, 15], the class to which FMS belongs to. However, no study reported high certainty evidence about the effects of antidepressants for pain, and for fibromyalgia just a moderate certainty exists, which has still to be interpreted with caution since many studies are tied to industries [16].

There are various types of antidepressant drugs each distinguished by their mechanism of action. A detailed description of each class would be useful.

### Tricyclic Antidepressants

Tricyclic antidepressants (TCAs) work by inhibiting noradrenaline and serotonin reuptake in the CNS, with some, like amitriptyline, also blocking voltage-gated sodium channels. This dual action is key to their success in treating pain. Amitriptyline, at 25 mg, has been found more effective than alternatives such as pregabalin and duloxetine in reducing pain intensity in FMS patients [17]. Despite this, studies on amitriptyline are older and involve fewer participants. Systematic reviews suggest it's more effective in treating sleep disturbances, fatigue, and improving quality of life compared to duloxetine and pregabalin [18], but not with a reduction in depressive symptoms [19]. Daily dosages for FMS pain management are lower than for depression treatment, suggesting serotonin-norepinephrine reuptake inhibitors may be better for patients with both FMS and depression. However, evidence still lack in this regard and reviews regarding TCA use for FMS remain inconclusive [16].

### Monoamine Oxidase Inhibitors

Monoamine oxidase (MAO) inhibitors, used in treating Parkinson's disease and depression, have been sparingly studied for FMS. Early studies showed that the second-generation MAO-A inhibitor, Moclobemide, didn't show significant analgesic activity in FMS compared to Amitriptyline or placebo [20, 21]. Another MAO-A inhibitor, Pirlindole, improved pain but didn't affect other symptoms, like asthenia or sleep quality [22]. Nonetheless, the evidence is still inconclusive.

### Selective Serotonin Reuptake Inhibitors (SSRIs)

SSRIs have been used for FMS treatment, but studies and reviews show inconsistent results [23]. Some found SSRIs significantly improved pain, sleep, depression, and quality of life [18]. However, a Cochrane meta-analysis and another review suggested that SSRIs are not superior to a placebo for primary FMS symptoms [16, 24].

Overall, it should be considered that serotonin has a bivalent activity on pain, with an alternate predominance of pronociceptive or antinociceptive effects, according to often unpredictable conditions [25]. For this reason, SSRI should not be considered as first line drugs for the treatment of depression in FMS.

### Serotonin and Noradrenaline Reuptake Inhibitors (SNRIs)

Currently, SNRIs are the most used drugs for FMS, with moderate efficacy evidence [16]. A meta-analysis showed 42% of SNRI-treated patients reported a 30% pain reduction, compared to 32% on a placebo [18]. However, nonresponders to SNRIs, particularly duloxetine, remain common. A study on 59 patients and 30 healthy controls found that nonresponders to SNRIs are characterized by less favorable metabolic parameters and comorbid depression and other psychiatric conditions [26]; however, it has been underlined by recent meta-analyses that duloxetine is most effective with FMS comorbid with depression [27].

**Duloxetine**, FDA-approved for FMS treatment, works by inhibiting serotonin and noradrenaline reuptake. Studies indicate a daily 120 mg dose improves pain and depression significantly [19]. Lower doses (30 mg) were less effective than pregabalin 450 mg and not superior to a placebo [17]. Higher doses (120 mg) are more effective but have more side effects, especially in cases with comorbid depression. Duloxetine's pain reduction effectiveness is independent of its mood effects and presence of major depressive disorders, but is more effective in FMS with depression [28]. Despite higher effective doses reported, we recommend starting with 30 mg daily, increasing dosage every two weeks.

**Milnacipran**, an SNRI with greater selectivity for norepinephrine, FDA-approved for FMS, shows promising improvements in pain, well-being, physical function, and fatigue at 100 mg and 200 mg per day dosages [29]. It also improves well-being and pain in FMS, excluding sleep quality, regardless of depression [30]. However, recent research indicates limited effectiveness in alleviating FMS symptoms [31]. Recommended dosage is 100 mg daily, starting with 12.5 mg and gradually increasing.

### Other Antidepressants

**Trazodone** is a Serotonin Antagonist and Reuptake Inhibitor (SARI) that acts as an antagonist of 5HT2a and 5HT2c serotonin receptors and inhibits serotonin reuptake [32]. At lower doses, it has hypnotic and anxiolytic effects [33], and has been used for FMS treatment. Trazodone (50-300 mg/day), alone or combined with pregabalin, notably improved sleep, reduced anxiety, depression, and Fibromyalgia Impact Questionnaire (FIQ) scores [34, 35]. However, 21.1% of patients reported tachycardia. In combination with pregabalin, it was better tolerated and improved FMS severity, depression, and pain.

**Reboxetine**, a Noradrenaline Reuptake Inhibitor (NRI), shows promise for chronic pain relief due to its selective noradrenaline activity. It has been reported to reduce musculoskeletal pain in patients with depression, independent of mood improvements [36]. Studies suggest that esreboxetine, reboxetine's active enantiomer, improves pain, fatigue, and FIQ scores at a 4 mg/day dosage [37]. A comparison study found no significant differences between reboxetine and amitriptyline in treating FMS symptoms [38].

**Mirtazapine** increases the release of serotonin and norepinephrine without inhibiting their reuptake. It has shown mixed results in FMS treatment. While some studies indicate improvements in pain, sleep disturbances, and quality of life [39–41], others suggest it's less effective than duloxetine [42]. A Cochrane review found mirtazapine may be useful for addressing FMS symptoms, but the evidence quality was low [43]. Adverse effects are common, and dosage adjustments may be needed to manage drowsiness.

## Anticonvulsants: Pregabalin and Gabapentin

Anticonvulsants like pregabalin and gabapentin, known as α2δ ligands, can impact nociception by modulating neuronal excitability [44]. They regulate calcium channels and therefore neurotransmitter release, including glutamate, noradrenaline, serotonin, dopamine, and substance P [45]. They may benefit FMS patients with neuropathic pain and psychiatric symptoms.

**Pregabalin** has shown effectiveness for FMS treatment and is FDA-approved. Studies found that a 450 mg/day dosage significantly reduced pain and improved sleep quality and fatigue [46]. This dosage's benefits on pain have been consistently confirmed [17]. A recent analysis found pregabalin 600 mg to be more effective for sleep and depression, while 150 mg primarily addressed fatigue and sleep but had no effect on pain [19]. Some patients on very low dosages (50 or 75 mg) for years experience low efficacy and long-term side effects, especially in patients with retinal nerve fiber layer damage [47]. A study found duloxetine 30-60 mg more effective than pregabalin 75-150 mg for FMS patients with depression [48]. Another study showed pregabalin had modest efficacy in pain relief, overall assessment, and function at 450 mg/day, and improved sleep at all dose levels but didn't provide consistent benefit at 300 and 600 mg/day [49]. The side effects of pregabalin, which increase with dosage, include dizziness, drowsiness, weight gain, and peripheral edema. It is recommended to initiate pregabalin treatment with a small dose (25–75 mg) to increase weekly. A controlled-release (CR) formulation of pregabalin, which can be administered once a day, is also an option [50].

**Gabapentin** showed analgesic activity in conditions with chronic neuropathic pain but results for FMS are limited [51]. Two randomized controlled trials (RCTs) investigating the use of gabapentin in FMS have been conducted, as analyzed in a Cochrane review; the reduction in pain was significantly greater in the gabapentin group (1200–2400 mg daily) compared to the placebo group [52]. However, this is based on low-quality evidence from a single trial. An extended-release gabapentin formulation improved pain and sleep in a small study of FMS patients [53]. In clinical practice, patients should begin with a low single dose of gabapentin (100–300 mg) before bedtime, and additional doses at breakfast and lunch after one week, followed by a predominantly evening dose increment. Adverse events associated with gabapentin are similar to those of pregabalin and include drowsiness, dizziness, fatigue, and weight gain.

## NMDR Antagonists

FMS is associated with increased N-methyl-D-aspartate receptor (NMDAR) activity, suggesting the potential of NMDAR modulation as a therapeutic intervention [54]. Ketamine, a non-competitive NMDAR antagonist administered intravenously, has been studied for its potential use in FMS treatment. In some studies, FMS patients receiving ketamine showed a progressive reduction in pain intensity during the infusion [55] and at 15 min post-infusion [56]. However, long-term analgesic effects of ketamine in FMS appear to be limited, potentially due to its pharmacokinetics [57]. Though single, low-dose, intravenous ketamine infusions provided only short-term reductions in self-reported pain intensity, higher doses and longer, more frequent infusions may offer better pain relief and more extended analgesia as suggested by two case studies [58, 59]. Despite the promise, it's crucial to monitor potential side effects including psychomimetic, gastrointestinal, and cardiovascular impacts. Further research is currently underway to understand the potential of ketamine in FMS treatment [60].

## Myorelaxants

**Cyclobenzaprine**, a muscle relaxant structurally similar to amitriptyline, has shown moderate effectiveness in FMS, with improvements in symptoms, particularly sleep quality [61]. Studies also report enhancements in sleep, fatigue, pain, and mood with very low-dose cyclobenzaprine (1–4 mg) taken at bedtime [62]. A recent trial with a sublingual formulation (TNX-102 SL, 5.6 mg final dose) showed improvements in daily pain, sleep quality, and FIQR by week 14 [63]. Common side effects include oral hypoesthesia and oral paresthesia, but can also cause nausea, weakness, constipation, and neurovegetative symptoms. Due to these side effects, dosage titration is important.

**Tizanidine** is a muscle relaxant with sedative properties (centrally acting alpha-2-adrenergic agonist). Considering the presence of α2-adrenergic receptors on the spinal synapse, where their activation cause inhibition of glutamate release, it is likely that tizanidine also has analgesic properties. Its effectiveness in FMS has been scarcely studied, but a trial involving 30 patients showed its potential in improving tender points and fatigue [64]. It may benefit patients with significant myofascial pain [65].

## Analgesics

Limited, low-quality evidence suggests NSAIDs are not effective for FMS treatment [66]. They may be used in mixed-type pain cases alongside traditional fibromyalgia medications.

### Opioids

Opioids can help manage chronic pain but may lead to tolerance, dependency, and side effects like constipation and sedation. Studies show decreased μ-opioid receptor availability in FMS, less improvement in pain-related interference with daily life (compared to tramadol), likelihood of opioid-induced hyperalgesia, and risk of opioid use disorder [67–69, 70]. Long-term opioid use can cause endocrine changes [71] and higher 10-year all-cause mortality rates [72]. Despite guidelines are against opioid analgesic use, they are often prescribed for FMS due to a lack of alternative treatments [73]. Their use should be considered only when all other pharmacological and non-pharmacological therapies have failed, or in low doses in combination with medications having a different mechanism of action. Cannabinoids should be preferred to opioids (see below).

**Tramadol**, a weak opioid and norepinephrine and serotonin reuptake inhibitor, showed efficacy in four randomized controlled trials for fibromyalgia treatment. A study found that tramadol at a dosage of 37.5 mg in combination with paracetamol significantly improved pain scores and FIQ results in comparison to a placebo group [74]. A systematic review with meta-analysis confirmed its positive effect on pain and quality of life in fibromyalgia patients [75]. In addition, the addictive potential of tramadol appears limited [76]. However, the quality of evidence in these studies was "low", and no significant improvements in depression or sleep quality were observed. In summary, the benefit-to-risk profiles of tramadol appears favourable, making tramadol a valuable consideration for managing cases of FMS where there is a significant pain component interfering with quality of life, or as an emergency ("SOS") medication in such scenarios.

**Tapentadol**, a centrally acting analgesic with dual action as a μ-opioid receptor agonist and a noradrenaline reuptake inhibitor, has potential for treating fibromyalgia due to its unique properties [77]. It offers similar analgesic effects to conventional opioids but with fewer side effects, although it does have a higher abuse potential than tramadol [78]. There is limited literature reporting its use in FMS patients. A study showed that it enhances conditioned pain modulation in a subpopulation of fibromyalgia patients, indicating increased endogenous pain inhibition [79]. Ideal dosages range from 50 to 250 mg twice daily for severe chronic pain, particularly with a prevalent neuropathic pain component [77, 80].

## GABAergic Drugs

GABA receptors, GABAa and GABAb, play a role in hyperpolarization and ion regulation [81]. GABAa receptors are ligand-gated ion channels selectively permeable to chloride ions, that entering inside the cell cause hyperpolarization. GABAb receptors are G-protein coupled receptors, with inhibitory effect on calcium current and activation of potassium current. GABAa are ligand-gated chloride channels, while GABAb are GTP-binding protein-coupled receptors and regulate K + and Ca2 + channels. Benzodiazepines, allosteric modulators of GABAa receptors, have been used for anxiety, sleep enhancement, and muscle relaxation. Although there is no conclusive evidence against these medications in the treatment of FMS due to lack of data [82, 83], recent clinical guidelines for FMS discourage their use due to their potentially harmful adverse effects [83], especially in combination with opioids [84]. As a result, their use is recommended just as add-on therapy. Short-acting benzodiazepines may help with initial insomnia. Non-benzodiazepine sedative-hypnotic medications, like zopiclone and zolpidem, can enhance sleep and alleviate fatigue. GABAb receptor is mostly concentrated in the spinal cord, and its agonists like baclofen and sodium oxybate have been used for motor neuron diseases and sleep disturbances. They showed effects on sleep, decreasing sleep onset latency and increasing slow wave sleep, in disorders like fibromyalgia [85].

## Antipsychotic Medications

There is some evidence that antipsychotic medications could be useful for FMS. **Quetiapine**, an antagonist at serotonin 5-HT2a and Dopamine D2 receptors, functions as a hypnotic at low doses (50 mg), an antidepressant at intermediate doses (300 mg), and an antipsychotic at high doses [86]. Its metabolite, norquetiapine, inhibits norepinephrine transporters and antagonizes α2 and 5-HT7 receptors [87]. Quetiapine, given in 25–100 mg daily doses, reduced stiffness and fatigue but not pain in FMS patients in one study [88]. A randomized, placebo-controlled study found it improved sleep but not other symptoms [89]. When used for patients with both depression and FMS, it significantly improved depression, pain, and quality of life [90], but less effectively than amitriptyline [91]. It may alleviate pain, sleep problems, depression, and anxiety in FMS patients with depression, but side effects like weight gain should be considered [92]. Use is suggested for FMS patients with major depression or persistent symptoms from depression/anxiety.

There is some evidence suggesting antipsychotics like **olanzapine** may help alleviate FMS symptoms, particularly alongside depression, but their use is limited by side effects such as weight gain and somnolence, which led some patients to discontinue treatment [93]. However, these findings are from older studies and further research is needed to confirm their effectiveness and safety [94].

## Cannabis and Cannabinoids

Cannabis sativa contains cannabinoids like THC and CBD that interact with specific receptors in the nervous and immune systems [95] and has been used to manage pain and other symptoms, like sleep [96]. Δ9-tetrahydrocannabinol (THC or Δ9-THC) has psychoactive effects [97] while cannabidiol (CBD) lacks psychoactive effects but demonstrated efficacy in addressing a wide range of conditions [98, 99], including chronic pain [100], Current research investigates both the whole plant and isolated cannabinoids (THC and CBD). Regarding isolated cannabinoids, [101], including two randomized controlled trials, concluded that there is no compelling, high-quality evidence to support the use of nabilone, a synthetic cannabinoid equivalent to THC, in treating FMS patients. Dronabinol, another form of synthetic THC, was evaluated in FMS patients with neuropathic pain [102]. Results showed a significant reduction in pain intensity and depression, leading to an improved quality of life, but only in patients able to tolerate the treatment (with an abandonment rate of about 25%) [102].

In terms of medical cannabis (MC), an observational study indicated reduced pain intensity from using cannabis preparations. An observational study, spanning six months and involving 367 FMS patients using MC preparations, revealed a reduction in the average pain intensity, while a small randomized placebo-controlled clinical study suggested THC-rich cannabis oil could help reduce symptoms and improve quality of life in FMS patients [103, 104]. In a recent publication, a prospective cohort study involving 30 women diagnosed with FMS demonstrated a significant improvement in various aspects of quality of life, including general quality of life, general health, physical health, and psychological well-being, after 30 days of cannabis treatment [105]. These findings suggest a potentially significant role for cannabis in the treatment of FMS, particularly in cases resistant to other treatments. Early cannabis treatment may lead to short-term benefits, improving quality of life by addressing pain, sleep, and physical and psychological well-being, although further studies are necessary to understand its potential and long-term impact. In particular, side effects include impacts on the central nervous system, psychological effects, vision, psychiatric issues, gastrointestinal problems, somnolence, diarrhea, psychological distress, and more [106]. Evidence supports avoidance of cannabis during adolescence and early adulthood, in people prone to or with mental health disorders, in pregnancy and before and while driving [106].

## Discussion and Treatment Guidelines

In the realm of FMS treatment, it has become increasingly clear that a solely pharmacological approach is not sufficient. The best results are achieved with a multidisciplinary approach, considering the complex and multifaceted nature of FMS. Various non-pharmacological therapies have been researched and tested, including balneotherapy [107], hyperbaric oxygen [107], and acupuncture [108] among others. However, while these treatments can be beneficial, pharmacological therapy remains a crucial component in managing fibromyalgia and achieving satisfactory outcomes.

The treatment of FMS and associated symptoms demands a personalized approach (Table 1). It should prioritize the use of antidepressants and anticonvulsants while generally excluding strong opioids, with specific exceptions for weak opioids like tapentadol and tramadol. It is imperative that pharmacological therapy be integrated within a multidisciplinary framework, aligning with the bio-psychosocial model, and complemented by physical and psychological interventions. Table 2 summarizes the main drugs used for FMS treatment and their initial and final dosages.

**Table 2:** List of the main drugs used for FMS in clinical practice. In italics those FDA-approved

**Drug**	**Drug family**	**Mechanism of action**	**Initial dose (mg)**	**Suggested maintenance dose (mg)**	**Evidence in FMS use**
*Duloxetine*	Antidepressant SNRI In the USA, it has been licensed for the treatment of fibromyalgia	Balanced inhibiton of NE and 5-HT reuptake Enhancement of the inhibitory descending pathway (PAG/spinal cord)	30	60–120	Widely used for FMS with moderate evidence of efficacy; Duloxetine, at a daily dose of 120 mg, is effective in improving both pain and depression, particularly in patients with FMS comorbid with depression. For clinical guidance, it is recommended to start Duloxetine at a lower dose of 30 mg in the morning, gradually increasing every two weeks up to the maximum dosage.
*Milnacipram*	Antidepressant SNRI In the USA, it has been licensed for the treatment of fibromyalgia.	Inhibition of NE and 5HT reuptake with a predominant effect on NE Enhancement of the inhibitory descending pathway (Locus Coeruleus/spinal cord)	12,5	50–200	May improve pain, well-being, fatigue, but not sleep quality. However, more research is needed in order to obtain more consistent data.
Mirtazapine	Antidepressant NaSSA: Noradrenergic and Specific Serotonin Antagonist	No effects on 5-HT and NE reuptake. The increase of the release of 5-HT and NE is due mirtazapine α2 –adrenergic antagonism. Normally, α2 –adrenergic receptors presynaptic receptors are inhibitory on 5-HT and NE release. 5-HT_3_ antagonism has inhibitory effect on GABA release from interneurons, which have a profound inhibitory effects on Ach, glutamate and NE release. Mirtazapine is an H1-Histamine receptor antagonist. A low dose, this effect could be exploited to treat insomnia.	15 mg	15 mg insomnia 30–45 mg depression	It has shown potential in reducing pain and sleep disturbances in FMS, with symptoms improving after 12 weeks of treatment. Data quality is low or very low. In real-life studies, it proved inferior to duloxetine in terms of efficacy and side effects.
Amitriptyline	Tricyclic Antidepressant (TCA)	NE and 5-HT reuptake inhibitor. Blockers of voltage-gated sodium channel. Antimuscarinic effects are largely responsible for ADR	5–25	30–60	It has demonstrated efficacy in managing FMS, notably through reducing pain intensity, enhancing sleep quality, and improving overall quality of life. However, as it does not significantly reduce depressive symptoms, for patients with coexisting FMS and depression, the use of SNRIs where analgesic and antidepressant dosages overlap, is recommended.
Trazodone	Antidepressant SARI: Serotonin Antagonist and Reuptake inhibitor	5-HT reuptake inhibitor and antagonist of 5-HT2a and 5-HT2c receptors. Trazodone acts like two different drugs, depending on the dose and formulation: at 150–600 mg it completely saturates Serotonin Transporter and acts as antidepressant; at 25 150 mg it maily retain antagonist action at α1 adrenergic and H1 histaminergic receptors with corresponding efficacy for insomnia	25–50 for insomnia 150 mg in divided dose	50–100 for insomnia (hypnotic/anxiolytic action) 150–375 extended release	It has shown effectiveness in FMS treatment, especially when combined with pregabalin, improving sleep quality and reducing anxiety, depression, and FMS severity. The antidepressant effects of trazodone appear only at moderate to high doses, whereas lower doses make trazodone a hypnotic and anxiolytic drug
Reboxetine	Approved Antidepressant in Europe, but not in the USA NRI: Selective Noradrenaline Reuptake inhibitor	Potentiation of noradrenergic descending pathways	4 mg in two doses	8 mg in two doses	It has a potential use for pain relief in patients with depression and chronic pain syndromes, but clinical data are still scarce.
*Pregabalin*	Anticonvulsant. In the USA, it has been licensed for the treatment of fibromyalgia	Ligand of the a2d subunit of voltage gated sodium channel. Modulation of calcium-channel expression in the spinal cord potentiation of the descending inhibitory pathways	25–75	150–600 die	It has been substantiated for FMS treatment, with studies demonstrating significant pain reduction and improvement in sleep quality and fatigue at dosages of 300 and 450 mg/day.
Gabapentin	Anticonvulsant	Ligand of the α2δ subunit of voltage gated sodium channel. Modulation of calcium-channel expression in the spinal cord potentiation of the descending inhibitory pathways	100–300	900–1600 die Up to 3200, if required	It is as a potential treatment option for FMS, particularly for patients who cannot use other treatments due to side effects, while acknowledging the limited and dated evidence base. Gabapentin, though primarily used for neuropathic pain conditions, has shown some promise in FMS with a significant reduction in pain at daily dosages of 1200–2400 mg.
Cyclobenzaprine	Myorelaxant	α2-adrenergic agonist Effects on motor neurons and in the dorsal horn of spinal cord	1–4 to 5–10	40–50	It has demonstrated moderate efficacy in improving symptoms in FMS patients, particularly enhancing sleep quality. It is advised to start with low-dose cyclobenzaprine (1–4 mg) at bedtime, then gradually increase to 5-10 mg over 14 weeks, while monitoring for potential side effects.
Tramadol	Opioid	µ agonist (weak) and 5-HT and NE reuptake inhibitor.	25–50	150	Tramadol has shown efficacy in FMS management with significant improvements in pain scores and quality of life, as supported by four randomized controlled trials. Consider tramadol for managing significant pain in FMS patients, mindful of its low quality of evidence and no observed improvements in depression or sleep quality
Tapentadol	Opioid	µ agonist (weak) and NE reuptake inhibitor	25–100	300–500	Tapentadol offers a unique therapeutic option with its dual mechanism of action, potentially beneficial for those patients with a prominent neuropathic pain component. Clinical guidelines suggest dosages from 50 to 250 mg twice daily, ideally 150 mg twice daily over at least three months, particularly for severe chronic pain with a prominent neuropathic component.
Alprazolam	Benzodiazepine	GABAa receptor PAM (Positive Allosteric Modulator). Anxiolitic effect	0.25–0.5	0–5-2.0	Due to potential harmful effects and an increased risk of severe interactions, especially with opioids, recent guidelines suggest limiting the use of benzodiazepines to add-on therapy for symptoms such as anxiety, sleep disturbances, and muscle stiffness.
Zolpidem	Z-drug	Non-benzodiazepine GABAa recepor modulator. Hypnotic	2,5–5	5–10	As for benzodiazepines, they should be employed as an add-on therapy to enhance sleep.
Quetiapine	Antipsychotic and mood stabilizer	Blocks Dopamine D2 recetors. Consider activating effects of dopamine on pain trasmission Mood stabilizer.	50–100 mg	100–200 mg	Quetiapine may have potential benefits in FMS patients, significantly improving sleep, depression, pain, and quality of life, particularly in those with comorbid major depression. However, physicians should consider quetiapine as a secondary treatment option due to its lower efficacy compared to amitriptyline and potential side effects such as weight gain, and recommend it only for FMS patients with comorbid major depression or unresolved depressive and/or anxious symptoms.
Cannabis	Phythopharmaceutical	Multiple effects on endocannabinoid system. Analgesic, anxiolytic, and sleep modulator	1 mg of THC, 1 mg/kg CBD	As tolerated, max 20 mg/kg CBD and 10-15 mg THC per day	Medical cannabis demonstrates potential for FMS treatment, with studies indicating significant reductions in pain intensity and improvements in quality of life. However, it should be used with caution in early adulthood, mental health disorders and before driving. Furthermore, its use depends on individual country legislation.

5-HT 5-hydroxytryptamine (serotonin); *ADR* Adverse drug reaction; *CBD* Cannabidiol; *FMS* Fibromyalgia syndrome; *NE* Norepinephrine; *PAG* Periaqueductal grey; *SNRI* Serotonin and Norepinephrine Reuptake Inhibitor; THC Delta-9-tetrahydrocannabinol

Furthermore, it's important to note that the efficacy of current FMS treatments is limited. To advance research in the field, there is a pressing need to develop new molecules and evaluate them in comprehensive, long-term comparative studies involving larger patient populations. While monotherapy would be the ideal therapeutic approach for FMS, it is often insufficient. Although combination therapies are commonly employed in clinical practice, there is a lack of substantial evidence supporting their actual benefit in FMS management. For instance, combinations involving drugs like pregabalin and antidepressants or amitriptyline have been studied in only a few trials. Given the limited effectiveness of monotherapy and the prevalent use of drug combinations, further well-designed prospective randomized clinical trials are necessary to explore the potential additive or synergistic effects of specific drug combinations tailored to individual patient needs (Table 1). This comprehensive, multidisciplinary approach is essential to addressing the complex nature of FMS.

High-quality evidence supports the effectiveness of several interventions in the management of fibromyalgia [27]. Cognitive-behavioral therapy demonstrates a short-term reduction in pain. Additionally, central nervous system depressants and antidepressants have shown benefits in pain management in the medium term. Similarly, antidepressants exhibit favorable results for improving the quality of life in the short term. However, it is worth noting that these improvements, while statistically significant, do not exceed the minimum clinically important change, which is 14 points on a 101-point scale for quality of life. Notably, evidence for long-term outcomes of these interventions is currently insufficient.

Further developments could arise from recognizing the heterogeneity of FMS as a factor limiting the effectiveness of pharmacological treatments. The existence of subgroups of FMS patients has been suggested by numerous studies, but most randomized controlled trials and studies on pathophysiology have not considered this variability factor, comparing the average results in the overall population of FMS patients. Analyzing the specific pathophysiological mechanisms of various patient subgroups could help identify drugs with a mechanism of action more targeted to the process that has led to the development of the disease. Help in this regard could come from recent studies on the polymorphism of genes for serotoninergic and dopaminergic system receptors, potentially useful for developing pharmacological strategies based on patient genotype.

The recognition that both biological (especially at the central nervous system level) and psychological and behavioral alterations are involved in the onset and maintenance of FMS suggests that, in addition to the use of symptomatic drugs, both cognitive-behavioral psychotherapy and appropriate physical exercise should be provided. The alliance between the patient and the doctor in identifying realistically achievable objectives with currently available therapeutic methods is a fundamental part of FMS treatment.

## Conclusions

In conclusion, there are currently various pharmacological options for FMS, including antidepressants, anticonvulsants, NMDA antagonists, myorelaxants, opioids, tramadol, tapentadol, benzodiazepines, antipsychotics, and cannabis. While all these drugs have demonstrated some benefits, the extent of these benefits is often limited. Specifically, they tend to either address only some domains of the complex FMS symptomatology or have a limited effect on pain. Additionally, it may be beneficial to divide patients into clinical subpopulations, such as FMS with comorbid depression, for more effective treatment. Furthermore, each treatment option comes with potential side effects and risks that necessitate careful consideration. It's crucial for future research to establish their effectiveness and safety profiles more definitively.

The hope of finding a way to cure the pain and other symptoms of FMS is still a dream, and we currently accept the view that a combination of pharmacological and non-pharmacological treatments is the best way to manage FMS. Hence, future studies should focus on identifying the best combination of pharmacological and non-pharmacological treatments to best address the different subgroups of patients.

## Data Availability

No datasets were generated or analysed during the current study.
